# Microtubule-Dependent Modulation of Adhesion Complex Composition

**DOI:** 10.1371/journal.pone.0115213

**Published:** 2014-12-19

**Authors:** Daniel H. J. Ng, Jonathan D. Humphries, Adam Byron, Angélique Millon-Frémillon, Martin J. Humphries

**Affiliations:** Wellcome Trust Centre for Cell-Matrix Research, Faculty of Life Sciences, University of Manchester, Manchester, M13 9PT, United Kingdom; King's College London, United Kingdom

## Abstract

The microtubule network regulates the turnover of integrin-containing adhesion complexes to stimulate cell migration. Disruption of the microtubule network results in an enlargement of adhesion complex size due to increased RhoA-stimulated actomyosin contractility, and inhibition of adhesion complex turnover; however, the microtubule-dependent changes in adhesion complex composition have not been studied in a global, unbiased manner. Here we used label-free quantitative mass spectrometry-based proteomics to determine adhesion complex changes that occur upon microtubule disruption with nocodazole. Nocodazole-treated cells displayed an increased abundance of the majority of known adhesion complex components, but no change in the levels of the fibronectin-binding α_5_β_1_ integrin. Immunofluorescence analyses confirmed these findings, but revealed a change in localisation of adhesion complex components. Specifically, in untreated cells, α_5_-integrin co-localised with vinculin at peripherally located focal adhesions and with tensin at centrally located fibrillar adhesions. In nocodazole-treated cells, however, α_5_-integrin was found in both peripherally located and centrally located adhesion complexes that contained both vinculin and tensin, suggesting a switch in the maturation state of adhesion complexes to favour focal adhesions. Moreover, the switch to focal adhesions was confirmed to be force-dependent as inhibition of cell contractility with the Rho-associated protein kinase inhibitor, Y-27632, prevented the nocodazole-induced conversion. These results highlight a complex interplay between the microtubule cytoskeleton, adhesion complex maturation state and intracellular contractile force, and provide a resource for future adhesion signaling studies. The proteomics data have been deposited in the ProteomeXchange with identifier PXD001183.

## Introduction

Adhesion complexes (ACs) serve as hubs to integrate and convey mechanical and chemical signals intracellularly and extracellularly [1], [2]. Upon integrin binding to the extracellular matrix (ECM), integrins cluster and recruit a large array of proteins. A literature-based study has identified in excess of 180 components potentially associated with ACs, termed the ‘adhesome’ [3], [4]. Some of these components tether the actin cytoskeleton to the plasma membrane [5], [6], some initiate signaling cascades [7]–[9], while others sense mechanical tension [10]–[13]. As such, ACs are involved in many cellular physiological activities including cell migration, ECM deposition and modification, cell differentiation and survival [1].

ACs are mechanosensitive and are regulated by tensional forces. The maturation of small nascent adhesions to large focal adhesions requires myosin II-mediated actomyosin contractile force [14], [15]. Conversely, suppression of myosin II activity by serum starvation [16] or pharmacological inhibition prevents the maturation of nascent adhesions [17]. On a molecular level, it has been shown that the application of force converts integrins from a relaxed state to a tensioned state and activates cellular signaling to FAK [11]. Furthermore, talin, a cytoplasmic binding partner of integrins, undergoes a conformational change upon the application of force to expose cryptic binding sites which allow binding of, and reinforcement with, vinculin [12]. Vinculin, in turn, is activated by the application of force via actin contractility and promotes the recruitment of AC proteins [18]. Together, these proteins act as a mechanosensing module that allows cells to respond rapidly to their environment by directly modulating the state of ACs in response to intra- or extracellularly applied forces. In contrast to focal adhesions, the formation of fibrillar adhesions is thought to occur via low-tensional forces due to the high translocation of α_5_β_1_-integrin complexes from the distal ends of FAs [19]. These α_5_β_1_-integrin complexes are rich in tensin, but lack other AC components such as α_v_β_3_-integrin, vinculin and paxillin, and display low levels of phosphotyrosine (pTyr) [20], [21]. It is clear that while tensional forces affect the different AC states, compositional differences also play an important role in determining the nature of the different AC states and their responses to tensional forces.

There is a complex cross-talk between microtubules, Rho GTPases, the tensional state of cells and ACs. Disruption of the cellular microtubule network hyperactivates RhoA-mediated myosin II contractility through the release and activation of microtubule-bound Rho guanine nucleotide exchange factor 2 (GEF-H1) [22], [23]. The increase in actomyosin contractility results in the assembly of stress fibres and focal adhesions [20], [24], [25]. In contrast, regrowth of the microtubule network stimulates the rapid activation of Rac1 and lamellipodial ruffling [26], [27], along with the targeting of focal adhesions by microtubules for disassembly [28]–[30]. These findings suggest that one of the physiological roles of microtubules is to regulate the turnover of ACs. Indeed, it has been shown in migrating cells that regulated disassembly of microtubules results in the spatiotemporal activation of RhoA via GEF-H1 at the leading edge and loss of GEF-H1 in cells results in altered microtubule organisation, impaired AC turnover and cell migration [31]. Therefore, microtubules tightly regulate AC formation, maturation and disassembly in a spatially and temporally controlled manner.

To address the molecular complexity of ACs, several groups have developed methods to isolate and define the composition of ACs using mass spectrometry (MS)-based proteomics [32], and in particular to characterise the global changes to the adhesome upon myosin II inhibition [33], [34]. These methods, coupled with downstream bioinformatics analysis, have identified novel AC regulators [32], compositional changes to ACs upon the loss of tension [33], [34], and highlighted negative regulators of AC maturation [34]. As such, MS-based proteomics is a powerful tool to characterise changes to protein complexes in a global, unbiased manner so as to derive biological hypotheses about complex biological systems.

While previous studies have established a complex cross-talk between microtubules and ACs [20], [26]–[30], the microtubule-regulated compositional changes to ACs have not been characterised in detail. In this study, we used a ventral plasma membrane isolation strategy to specifically target ACs for MS analysis. We found that upon microtubule disruption, there was an intracellular force-dependent switch in the maturation state of ACs towards focal adhesions, such that α_5_-integrin is redistributed to focal adhesions from other ACs, and is accompanied by an overall increase in the abundance of AC components. In summary, we present a novel MS dataset which catalogs the compositional changes to ACs upon microtubule disruption in a global, unbiased manner, which sheds light on the complex cross-talk between microtubules, intracellular forces and ACs.

## Materials and Methods

### Reagents

Ligands used for adhesion assays were bovine plasma fibronectin (FN), and poly-D-lysine (PDL) and were purchased from Sigma-Aldrich. Dimethyl 3,3′-dithiobispropionimidate (DTBP) was purchased from Thermo Scientific. InstantBlue was purchased from Expedeon; acetonitrile (ACN, CHROMASOLV Plus), formic acid (FA), dithiothreitol (DTT) and iodoacetamide (IA) were purchased from Sigma-Aldrich. Sequencing grade trypsin was purchased from Promega.

### Antibodies

Primary antibodies used in this study for Western blotting were specific for α_5_-integrin (H-104 rabbit polyclonal antibody, Santa Cruz Biotechnology), α_v_-integrin (rabbit polyclonal antibody, Abcam), talin (8D4 mouse monoclonal antibody, Sigma-Aldrich), vinculin (hVIN-1 mouse monoclonal antibody, Sigma-Aldrich), paxillin (clone 349 mouse monoclonal antibody, BD Biosciences), ILK (EPR1592 rabbit polyclonal antibody, Abcam), PDLIM5 (rabbit polyclonal antibody, Abcam), filamin A (PM6/317 mouse monoclonal antibody, Abcam), ELKS (ELKS-30 mouse monoclonal antibody, Sigma-Aldrich), transferrin receptor (H68.4 mouse monoclonal antibody, Life Technologies) and BAK (rabbit polyclonal antibody, Sigma-Aldrich). Primary antibodies used in this study for immunofluorescence experiments were specific for α_5_-integrin (mAb11 rat monoclonal antibody), tubulin (YL1/2 rat monoclonal antibody, Millipore), vinculin (FITC-conjugated hVIN-1 mouse monoclonal antibody, Sigma-Aldrich), α_v_β_3_-integrin (LM609 mouse monoclonal antibody, Millipore), tensin-1 (H-300 rabbit polyclonal antibody, Santa Cruz Biotechnology), pTyr (P-Tyr-100 mouse monoclonal antibody) and fibronectin (F3648 rabbit polyclonal antibody, Sigma). FITC-, Texas Red- or Alexa Fluor 647-conjugated phalloidin (Life Technologies) were used to immunostain actin filaments. Secondary antibodies for the Odyssey imaging system (LI-COR Biosciences) used in this study were Alexa Fluor 680- or 800-conjugated donkey polyclonal antibodies specific for rat, mouse or rabbit IgG. Secondary antibodies for immunofluorescence experiments were Cy2-, Cy3- or Cy5-conjugated donkey monoclonal antibodies specific for rat, mouse or rabbit IgG.

### Cell culture

Human foreskin fibroblasts (HFFs) were obtained from ATCC and grown in Dulbecco's modified Eagle medium (DMEM), supplemented with 10% (v/v) fetal calf serum (Lonza), 2 mM L-glutamine, penicillin and streptomycin and incubated at 37°C and 5% (v/v) CO_2_. For all cell-based assays, cell-bound ECM was removed by trypsinising cells, washing twice with phosphate-buffered saline without Ca^2+^ and Mg^2+^ (PBS^−^), resuspending cells in DMEM and incubated at 37°C and 5% CO_2_ (v/v) for 30 minutes. 1×10^3^ to 2×10^3^ cells were plated on ligand-coated MatTek dishes or 1.25 to 1.50×10^6^ cells were plated on ligand-coated 10 cm-diameter plastic tissue culture dishes for 16 hours and incubated at 37°C and 8% CO_2_ (v/v) in serum-free conditions.

### Isolation of ventral plasma membrane complexes

**Figure 1:**
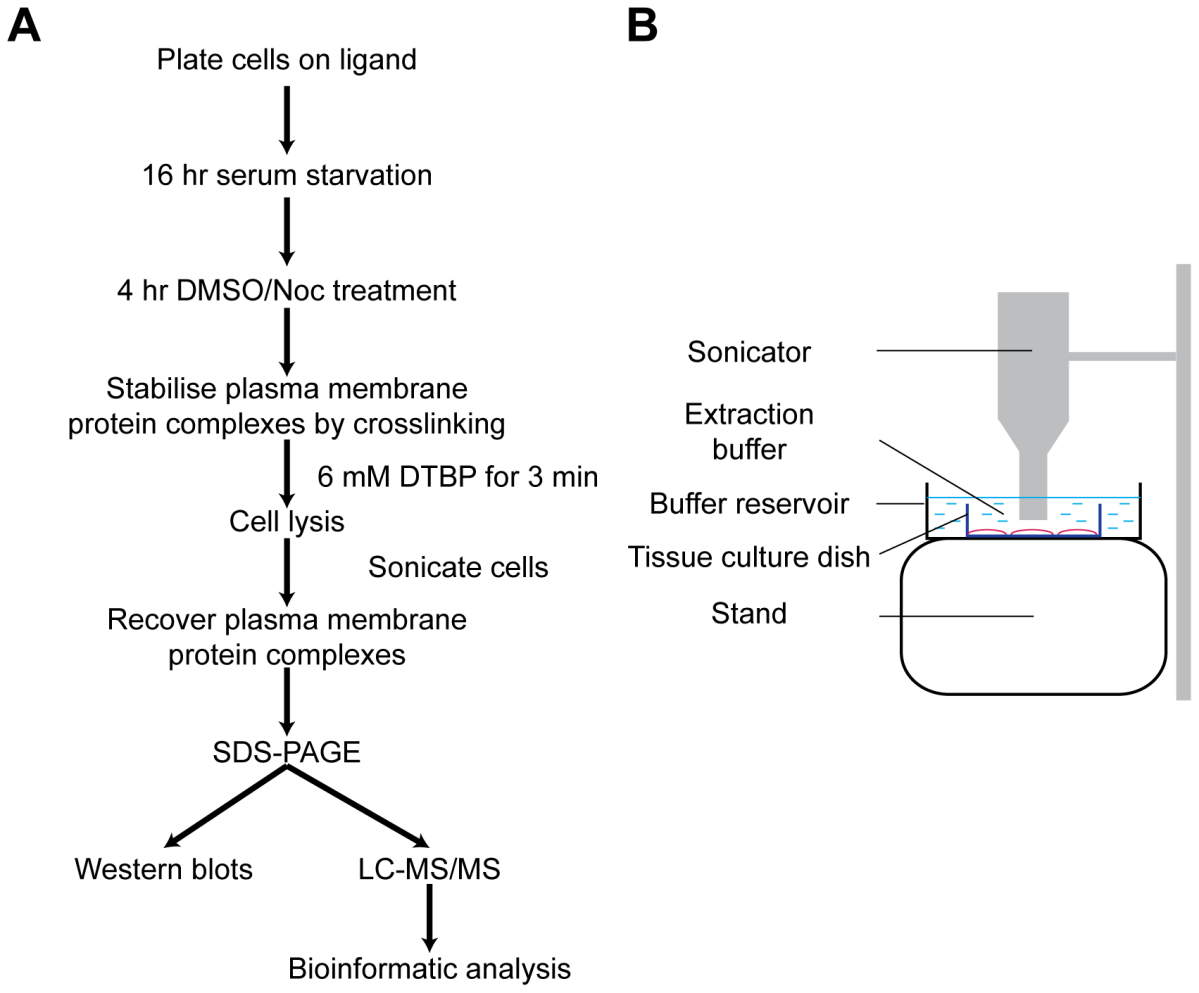
Isolation of ventral plasma membrane complexes. A) Workflow for the MS-based proteomics analysis of isolated ventral plasma membrane complexes. B) Schematic of the method to lyse cells after chemical cross-linking.

Isolation of ventral plasma membrane complexes was carried out using a similar approach to previously described studies [32]–[34] and as outlined in Fig. 1. Briefly, HFFs were serum-starved for 16 hours and then treated with DMSO or nocodazole for 4 hours. Cells were cross-linked with 6 mM DTBP for 3 minutes and quenched with Tris-HCl, pH 8.5 (Fig. 1a). Cross-linked cells were permeabilised with ice-cold extraction buffer (20 mM NH_4_OH, 0.5% Triton X-100 in PBS^−^) followed by sonication with a VibraCell VCX 500 (Sonics & Materials) for 2.5 minutes to lyse cells (Fig. 1b). Ventral plasma membrane complexes were collected in reducing Laemmli sample buffer (250 mM Tris-HCl, 40% (w/v) glycerol, 8% (w/v) sodium dodecyl sulfate, 0.02% (w/v) bromophenol blue and 10% (v/v) β-mercaptoethanol) and heated at 95°C for 10 minutes to denature proteins and reduce DTBP. Ventral plasma membrane complexes samples were fractionated by SDS-PAGE and used either for Western blotting or visualised with InstantBlue to be used for in-gel proteolytic digestion.

### In-gel proteolytic digestion

In-gel proteolytic digestion was carried out as described by Humphries *et al.* [32]. Briefly, gel lanes were cut into five slices, and each slice cut into ∼1 mm^3^ pieces. Gel pieces were destained with 50% (v/v) ACN in 12.5 mM NH_4_HCO_3_, dehydrated with ACN, reduced in 10 mM DTT, alkylated in 55 mM IA, washed with alternating washes of 25 mM NH_4_HCO_3_ and ACN, dehydrated and digested with trypsin (12.5 ng/µl). Peptides were collected in one wash of 99.8% (v/v) ACN and 0.2% (v/v) FA and one wash of 50% (v/v) ACN and 0.1% (v/v) FA.

### MS data acquisition and processing

LC-MS/MS was carried out using an UltiMate 3000 Rapid Separation LC (Dionex Corporation) coupled to an Orbitrap Elite (Thermo Fisher Scientific) mass spectrometer. Peptides were concentrated on a pre-column (20 mm×180 µm i.d., Waters) and separated on an analytical column (250 mm×75 µm i.d., 1.7 µm BEH C18, Waters) using an increasing ACN gradient, with a starting mixture of 1% (v/v) of 0.1% FA in ACN and gradually brought up to 25% (v/v) of 0.1% FA in ACN over 45 minutes at 200 nl/min. Peptides were selected for fragmentation automatically by data-dependent analysis.

Peak list files were searched against a modified version of the IPI Human database (version 03_70_4_0, containing 87,084 proteins) using an in-house Mascot server (version 2.2.06, Matrix Science). Fixed modification was set for carbamidomethylation of cysteine and variable modification was set for oxidation of methionine. Maximum missed cleavages for tryptic peptides was set to one. Only monoisotopic precursor ions that were doubly or triply charged were considered. Results were loaded in Scaffold (version, 3.6.5, Proteome Software), and peptide and protein identification threshold was set to 95% and 99% confidence, respectively. Data were exported as a Samples Report and analysed in Excel (Microsoft). The unweighted spectral count per protein was first normalised against the total spectra, and then normalised against the molecular weight of each protein.

The MS proteomics data were deposited to the ProteomeXchange Consortium [35] via the PRIDE partner repository with the dataset identifier PXD001183 and DOI 10.6019/PXD001183.

### Hierarchical clustering and gene ontology analysis

Hierarchical clustering was performed using Cluster 3.0 (C Clustering Library, version 1.37) and MultiExperiment Viewer (TM4 Microarray Software Suite). Normalised spectral counts were multiplied by 1000 and clustered using uncentred Pearson correlation as the distance metric and average linkage as the linkage criteria. Protein lists with IPI accession numbers were searched using the functional annotation tool in DAVID [36], [37]. To plot pie charts, −log_10_(Bonferroni-corrected *p*-value) was calculated for each gene ontology term.

### Protein-protein interaction network

Cytoscape (version 2.8.3) was used to visualise protein-protein interactions networks from MS data. The human interactome used was built from the Protein Interaction Network Analysis platform *Homo sapiens* network (release date, 28 June 2011) and *Mus musculus* network (release date, 28 June 2011) [38], the ECM interactions database MatrixDB (release date, 26 August 2010) [39], and a literature-curated database of integrin-based adhesion-associated proteins [3], [4]. Protein nodes were coloured according to the log_2_(nocodazole/DMSO) values. Nodes were sorted based on their cellular localisation using ‘cellular component’ gene ontology terms and manually validated to reduce discrepancies in assignments. Cytoplasmic nodes were further ordered according to the interaction-network distance away from the plasma membrane integrins, i.e. 1-hop, 2-hop. Node size was adjusted to reflect the molecular weight-normalised spectral counts.

### Immunofluorescence experiments

HFFs were serum-starved for 16 hours and then treated with DMSO or nocodazole for 4 hours. To visualise microtubules, cells were fixed with 4% (w/v) paraformaldehyde supplemented with 0.05% (v/v) glutaraldehyde for 7 minutes at room temperature. For all other immunofluorescence experiments, cells were fixed with 4% (w/v) paraformaldehyde for 7 minutes at room temperature. Cells were permeabilised with 0.2% (v/v) Triton X-100 for 10 minutes at room temperature, blocked with 1% (w/v) bovine serum albumin (BSA) supplemented with 0.01% (v/v) Tween 20 (BSA-T) for 30 minutes and incubated with primary antibody diluted in BSA-T for 1 hour at room temperature. Cells were washed with PBS^−^ three times, incubated with secondary antibody diluted in BSA-T for 1 hour at room temperature, and washed with PBS^−^ three times. Immunostained cells were imaged with a Delta Vision [RT] (Applied Precision) restoration microscope using a [60x/1.42 Plan Apo/Oil immersion] objective and the Sedat Quad filter set (Chroma [89000]). The images were collected using a Coolsnap HQ (Photometrics) camera on the Softworx software (Applied Precision) as a single stack and analysed using ImageJ (version 1.48c) National Institutes of Health).

## Results

### Methodology to isolate ventral plasma membrane complexes for the characterisation of ACs upon microtubule disruption

To characterise the global changes to ACs upon the loss of microtubules, we adapted previously described workflows to allow the specific isolation of ventral membrane protein complexes for analysis by MS-based proteomics [32]–[34]. Ventral plasma membrane complexes from cells adherent to fibronectin (FN) or a control ligand, poly-D-lysine (PDL), were stabilised by cross-linking, isolated after cell lysis using sonication (Fig. 1), and analysed either by Western blotting or MS-based proteomics.

**Figure 2:**
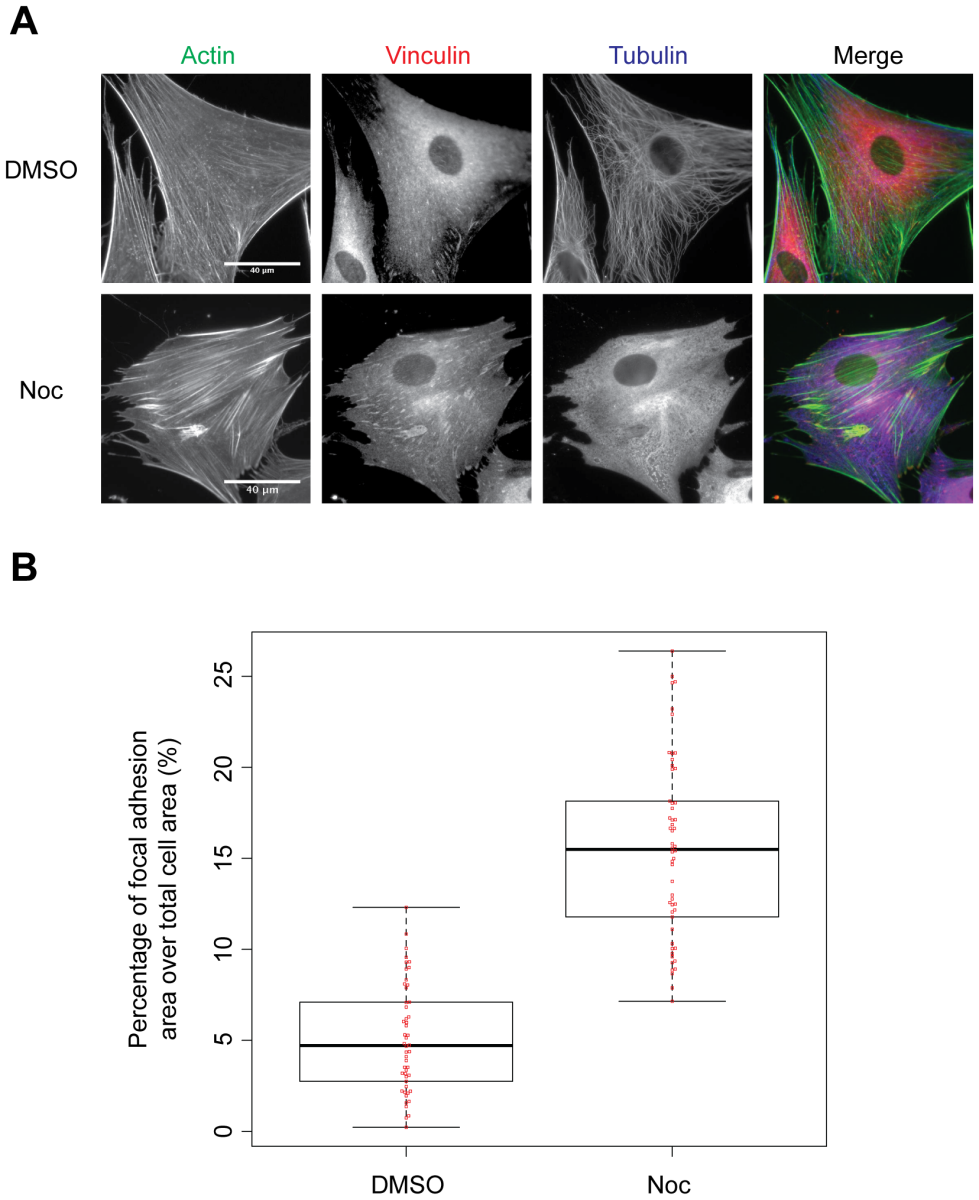
Changes to actin stress fibres and vinculin-containing ACs upon microtubule disruption. Serum starved HFFs were treated with DMSO and nocodazole (Noc) for 4 hours and fixed either with A) paraformaldehyde supplemented with glutaraldehyde to preserve microtubules, and immunostained for actin (green), vinculin (red) and tubulin (blue) or with B) paraformaldehyde and immunostained for vinculin. Vinculin staining was quantified as a percentage of the total cell area and displayed as a Beeswarm-Boxplot (line, median; box, interquartile range; whiskers, maximum and minimum; *n* = 51 to 55 cells).

Initially, serum-starved HFFs adherent to FN were treated with nocodazole to disrupt the microtubule network, and fixed and immunostained for actin, vinculin and tubulin (Fig. 2a). In agreement with previous studies [8], [20], [24], serum-starved vehicle-treated cells displayed an elongated cell morphology, a well-formed microtubule network, a sparse and disorganised actin cytoskeleton and small peripheral vinculin-containing ACs. In contrast, nocodazole-treated cells displayed a contracted morphology with distinctive ‘finger-like’ protrusions at cell edges, loss of the microtubule network, dense and thick actin stress fibres and large, vinculin-containing ACs throughout the cell (Fig. 2a). Quantification of vinculin-containing ACs showed a 3.3-fold increase in area in nocodazole-treated compared to vehicle-treated cells (Fig. 2b).

**Figure 3:**
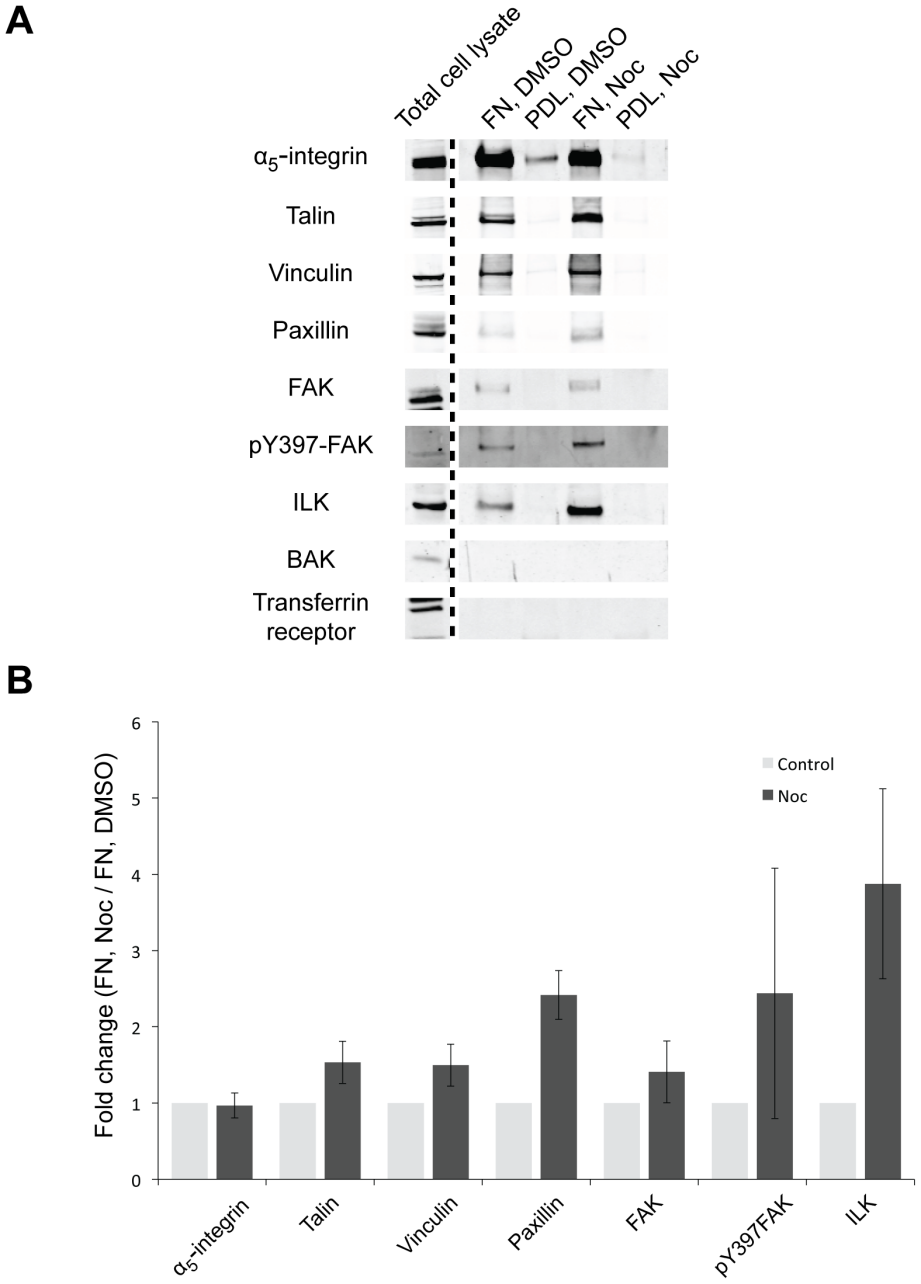
Changes in isolated ventral plasma membrane complexes upon microtubule disruption. A) Serum-starved HFFs plated on either fibronectin (FN) or poly-D-lysine (PDL) were treated with DMSO or nocodazole (Noc) for 4 hours, and ventral plasma membrane complexes were isolated for Western blotting for AC components (α_5_-integrin, talin, vinculin, paxillin, FAK, pY397-FAK and ILK) and non-AC components (BAK and transferrin receptor). B) Protein bands were quantified and normalised to FN, DMSO (mean ± SD; *n* = 3).

To determine if changes to ACs upon loss of microtubules could be detected from isolated ventral plasma membrane complexes, complexes were isolated from cells adherent to FN or PDL following treatment with vehicle (DMSO) or nocodazole, and analysed by Western blotting (Fig. 3). In agreement with immunofluorescence results, ventral plasma membrane complexes from nocodazole-treated cells exhibited a general increase in abundance in AC components compared to vehicle-treated cells: 1.5-fold increase in vinculin, 1.5-fold in talin, 2.4-fold in paxillin, 1.4-fold in FAK, 2.4-fold in phosphotyrosine-397 FAK (pY397-FAK), and a 3.8-fold increase in ILK (Fig. 3). Notably, the abundance of α_5_-integrin did not change in nocodazole-treated cells compared to vehicle-treated cells (Fig. 3). This general increase in the abundance of AC components is in agreement with previous studies that showed the assembly and growth of focal adhesions upon the loss of microtubules [20], [24], [25]. As controls, the non-AC components transferrin receptor and BAK were not detected in ventral plasma membrane complexes from cells adhered on FN (Fig. 3a). Moreover, AC components were not enriched in ventral plasma membrane complexes isolated from cells adherent to PDL (Fig. 3a). Taken together, these results validate this method for the specific isolation of ACs to detect quantitative protein changes upon the loss of microtubules.

### Quantitative MS analysis of isolated ventral plasma membrane complexes upon microtubule disruption

**Figure 4:**
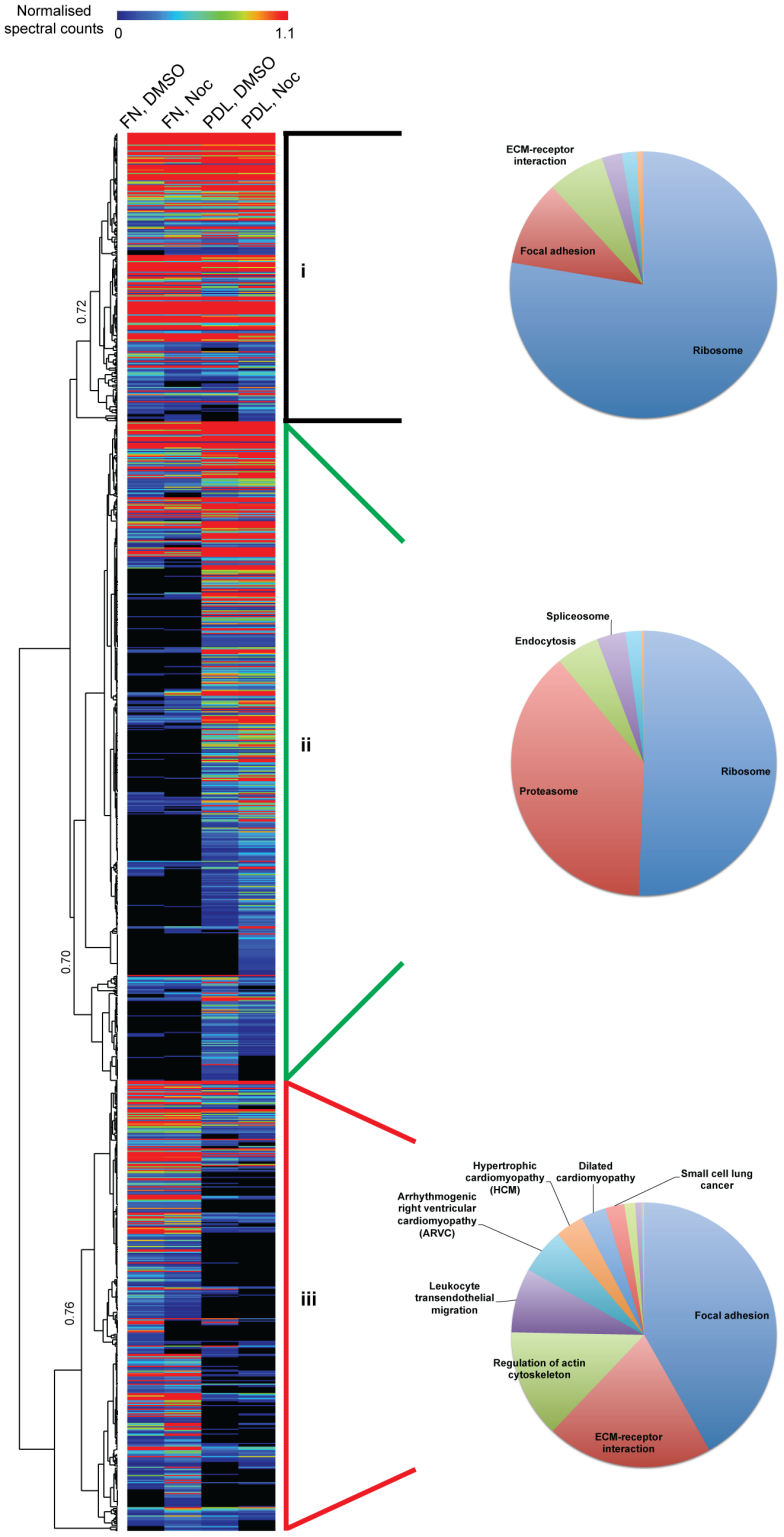
Unsupervised hierarchical clustering analysis of ventral plasma membrane complexes upon microtubule disruption. Complete output of unsupervised hierarchical clustering analysis of proteins identified by MS. Correlations at selected dendrogram nodes are indicated. Three clusters were identified: i) proteins present in equal abundance in both fibronectin (FN) and poly-D-lysine (PDL) samples; ii) proteins enriched in PDL; and iii) proteins enriched in FN. Clusters were subjected to pathway enrichment analysis and significantly enriched KEGG pathway terms (Bonferroni-corrected *p*-value<0.05) are displayed as pie charts.

To analyse the changes to ACs upon the loss of microtubules in a global, unbiased manner, ventral plasma membrane complexes from vehicle- and nocodazole-treated cells were analysed by quantitative MS, using spectral counting as a measure of relative protein abundance. MS identified 850–1000 proteins in each sample (S1 Table). To identify patterns in the relative abundances of different proteins in each condition, hierarchical clustering was performed (Fig. 4). Three main clusters were identified: (i) proteins of similar abundance in both FN and PDL samples, (ii) proteins enriched to PDL, and (iii) proteins enriched to FN. KEGG pathway analysis revealed that the majority of significantly enriched functional terms (Bonferroni-corrected *p*-value<0.05) for proteins enriched to FN (cluster iii) were AC-related (i.e. focal adhesion, ECM-receptor interaction, regulation of actin cytoskeleton and leukocyte transendothelial migration) (S2 Table). Other terms enriched to FN (cluster iii) were related to diseases that have been shown to be associated with dysfunctional adhesion complexes i.e arrhythmogenic right ventricular cardiomyopathy, hypertrophic cardiomyopathy, dilated cardiomyopathy, and small cell lung cancer [40]. In contrast, the most significantly enriched terms for proteins of similar abundance in FN and PDL (cluster i) and proteins enriched to PDL (cluster ii) were non-AC related (i.e. ribosome and proteasome). Therefore, hierarchical clustering confirmed a global enrichment of AC-related proteins to cells spread on FN.

**Figure 5:**
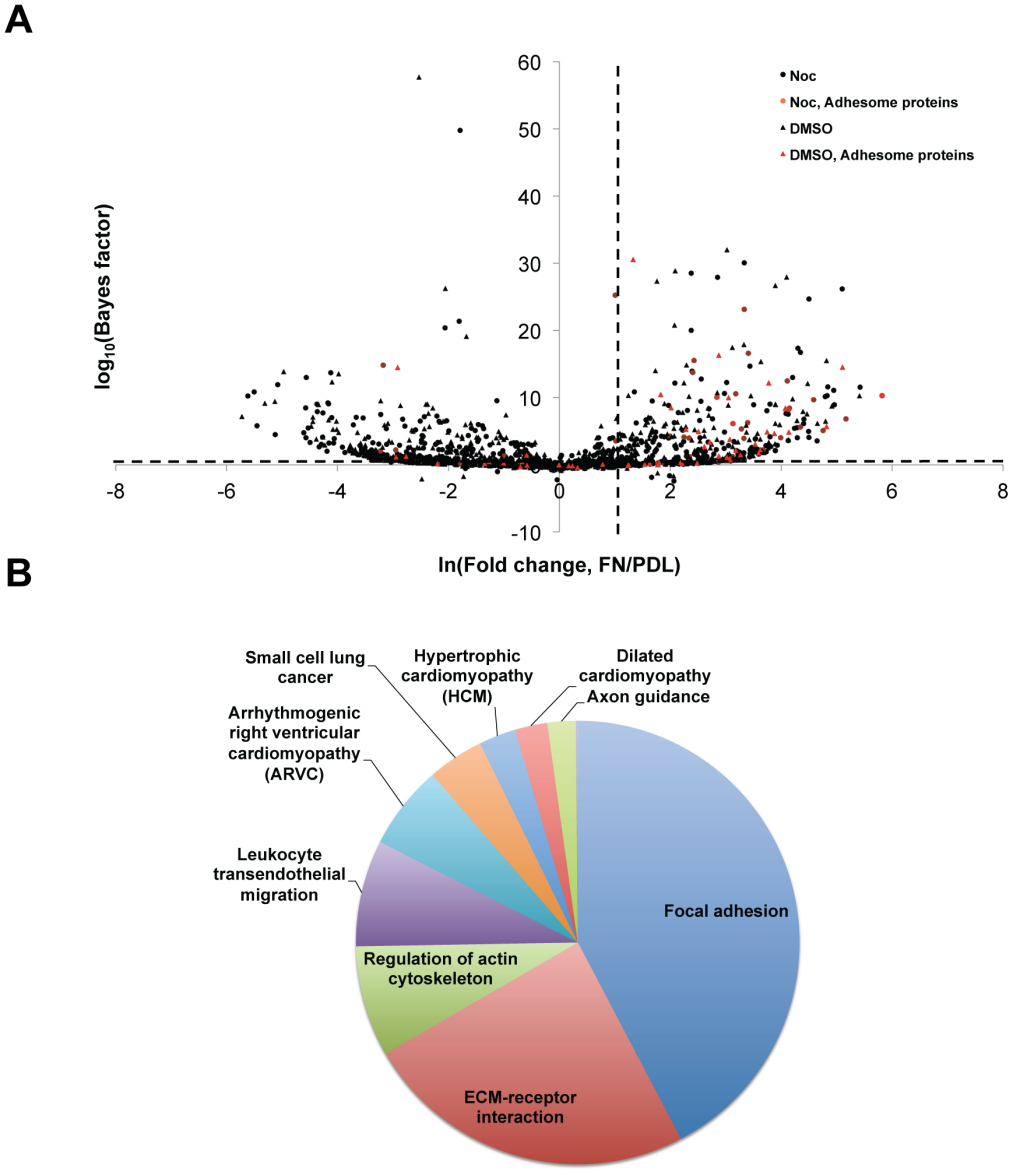
Identification of statistically enriched adhesion complex proteins. Volcano plot of QSpec output, log_10_(Bayes factor) and ln(fold change, FN/PDL). Horizontal dashed line corresponds to Bayes factor of 10 and vertical dashed line corresponds to a fold change (FN/PDL) of 3. Proteins statistically enriched to FN lie in the top right quadrant (log_10_(Bayes factor) ≥1, and ln(fold change, FN/PDL) ≥1.1), which represents a conservative false discovery rate estimate of less than 5% [41]. Proteins statistically enriched to FN were subjected to pathway enrichment analysis and significantly enriched KEGG pathway terms (Bonferroni-corrected *p*-value<0.05) are displayed as a pie chart. FN, fibronectin; PDL, poly-D-lysine.

To refine the dataset further, QSpec [41] was used to identify proteins that were statistically enriched in FN over PDL. QSpec is a statistical framework for spectral counting data, which gives the statistical measure of proteins that are differentially expressed in two conditions. A total of 196 proteins were statistically enriched to FN over PDL, of which 26 proteins were found in the literature-curated adhesome [3] (Fig. 5a). In agreement with hierarchical clustering data in Fig. 4 cluster (iii), the most significantly enriched terms for the 196 proteins were AC-related (Fig. 5b and S2 Table).

**Figure 6:**
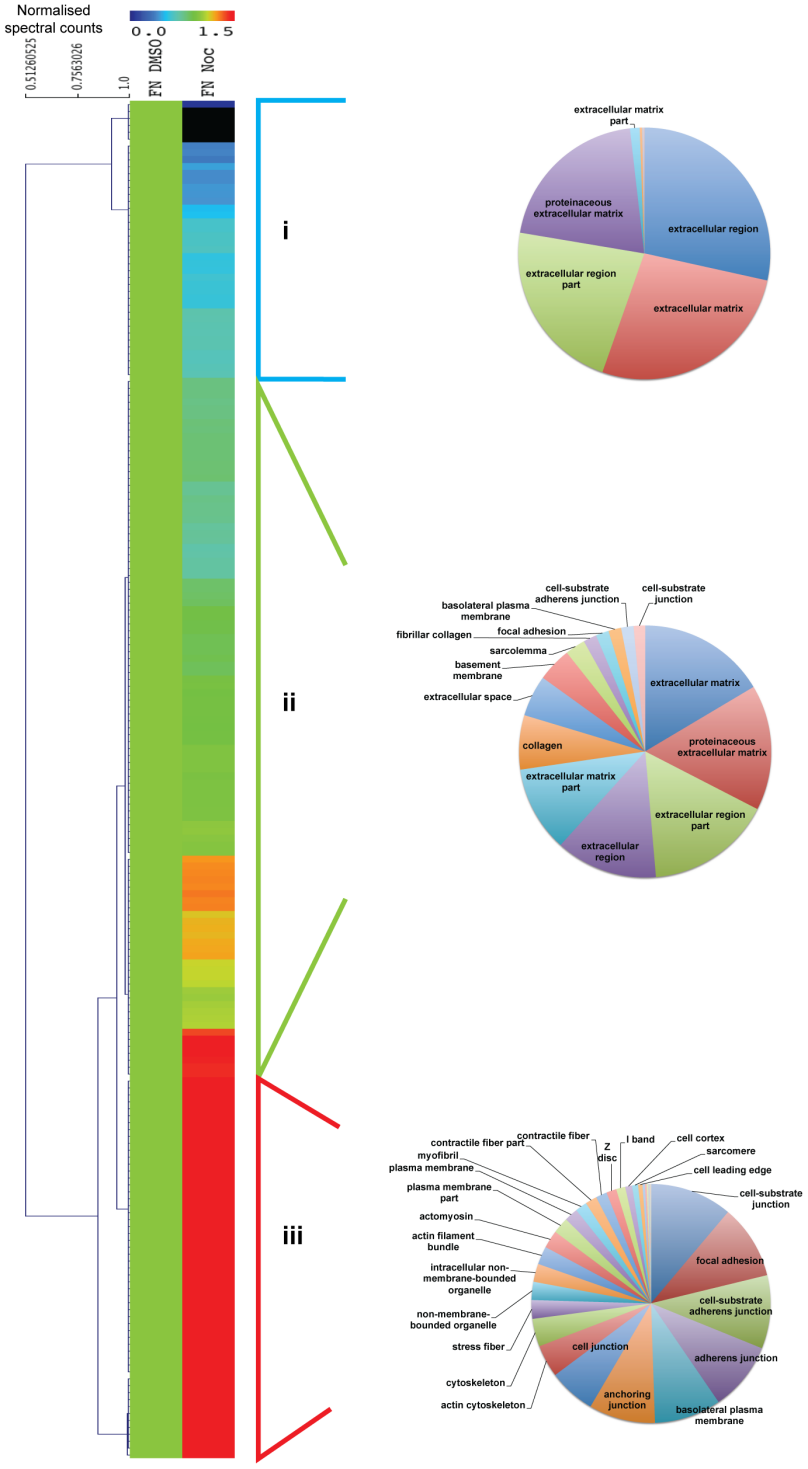
Unsupervised hierarchical clustering analysis of proteins statistically enriched to FN. Heat map dendogram displaying the hierarchical clustering of the fold change of FN, Noc relative to FN, DMSO. Three clusters were identified and analysed by gene ontology enrichment analysis to identify significantly enriched ‘cellular component’ terms (Bonferroni-corrected *p*-value<0.05). In general, the three clusters were: i) proteins that decreased in abundance upon nocodazole treatment and were enriched in ECM-related terms; ii) proteins that did not change and were enriched in ECM-related terms; and iii) proteins that increased and were enriched in AC-related terms. FN, fibronectin; PDL, poly-D-lysine; Noc, nocodazole.

To assess the relative changes of proteins statistically enriched to FN upon the loss of microtubules, the fold change of normalised spectral counts of AC components identified from nocodazole-treated cells relative to those from vehicle-treated cells were calculated. Hierarchical clustering was used to provide an overview of the relative changes to proteins (Fig. 6). Three clusters were observed upon the loss of microtubules: (i) proteins that decreased in abundance, (ii) proteins that did not change in abundance, and (iii) proteins that increased in abundance. Gene ontology enrichment analysis of these clusters using ‘cellular component’ terms revealed that the majority of significantly enriched terms for clusters (i) and (ii) were related to the ECM. In contrast, the majority of significantly enriched terms for cluster (iii) was related to cell adhesion, cell junctions and associated structures (Fig. 6 and S2 Table). All the AC proteins that were enriched in FN over PDL conditions as identified by Western blotting were found in cluster (iii) (Fig. 3).

In summary, we have isolated ACs upon microtubule disruption and performed MS and clustering analysis to identify adhesion proteins regulated by microtubules. These findings demonstrate that microtubule disruption results in a general increase in the abundance of AC components, whereas ECM proteins remain the same or are decreased upon microtubule disruption.

### Protein-protein interaction network analysis

**Figure 7:**
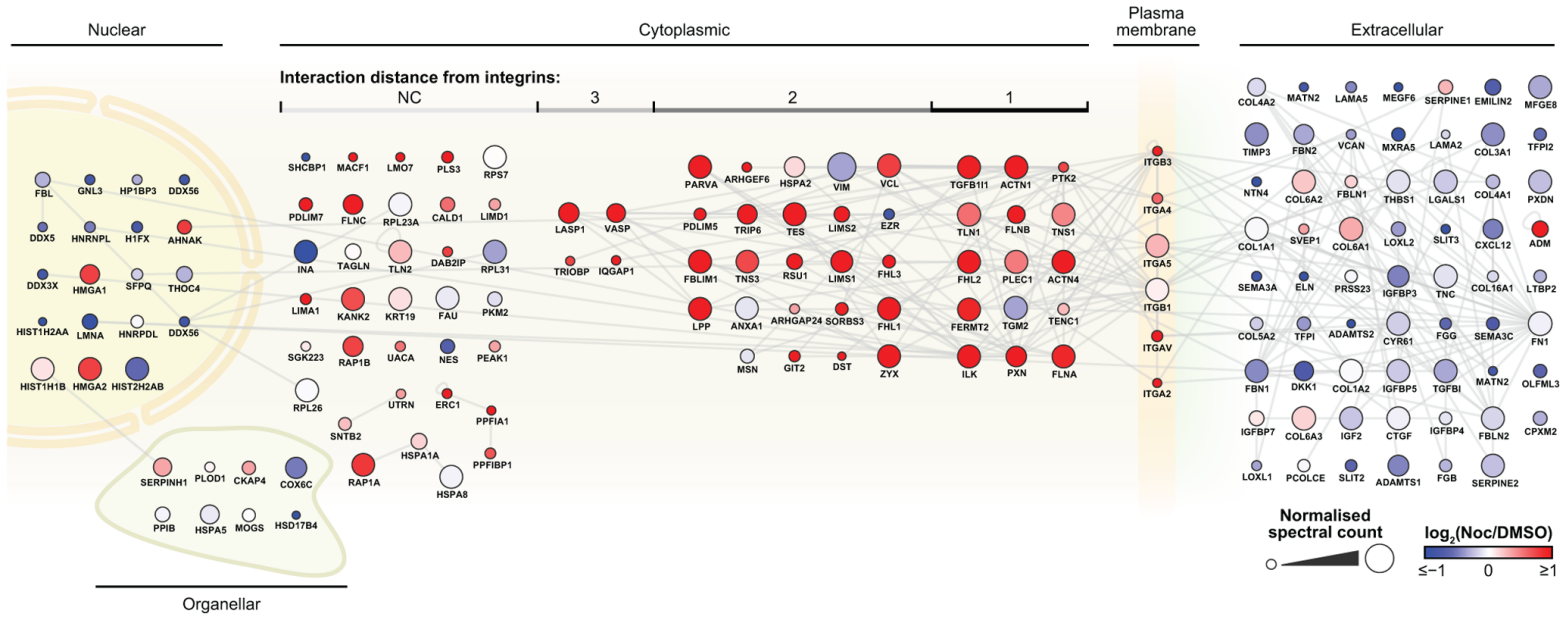
Protein-protein interaction network analysis of FN-enriched ventral plasma membrane complexes. Node colour was determined by a blue-red colour gradient corresponding to the log_2_(Noc/DMSO) values, with red nodes indicating an increase and blue nodes a decrease in protein abundance upon nocodazole treatment. Nodes were sorted according to their cell localisation and node size was proportional to the normalised spectral counts. The network was ordered according to the interaction network distance relative to the plasma membrane integrins, 1-hop, 2-hop, etc. Corresponding gene names are displayed underneath each node for clarity. FN, fibronectin; Noc, nocodazole.

To interrogate further the biological function of microtubule-induced AC changes, proteins statistically enriched to FN were mapped onto a protein-protein interaction network (PPI) and were arranged according to their cellular localisation (extracellular, plasma membrane, cytoplasmic, organelle and nucleus) (Fig. 7). To reduce the complexity of the interactions between cytoplasmic proteins, the network was ordered according to the interaction network distance relative to the plasma membrane-intercalated integrins. Protein nodes were coloured according to their change in abundance in nocodazole- relative to vehicle-treated conditions (red indicating an increase and blue a decrease in protein abundance upon nocodazole treatment). Upon nocodazole treatment, the majority of proteins in the ECM decreased, whereas plasma membrane and cytoplasmic proteins increased (Fig. 7). Although the majority of plasma membrane integrins increased upon microtubule disruption, the main FN-binding α_5_ and β_1_ integrins remained relatively unchanged, consistent with Western blotting results (Fig. 3).

**Figure 8:**
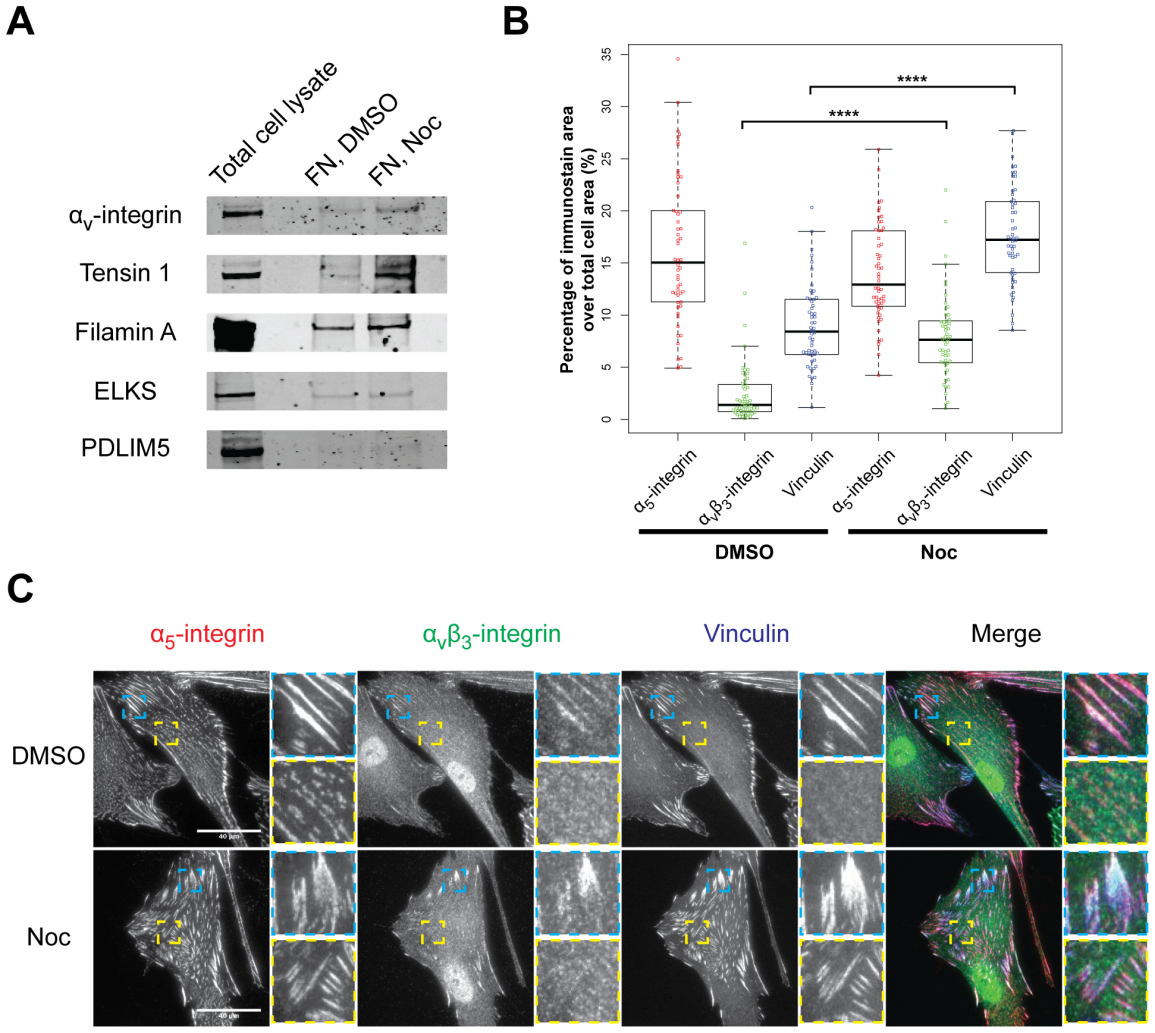
Validation of changes in AC components observed by MS. A) Isolated ACs were probed by Western blotting for α_v_-integrin, tensin-1, filamin A, ELKS and PDLIM5. B) To investigate the pattern of changes to integrins upon loss of microtubules further, HFFs treated with DMSO or nocodazole (Noc) were fixed and immunostained for α_5_-integrin, α_v_β_3_-integrin and vinculin and quantified as a percentage of the total cell area (line, median; box, interquartile range; whiskers, maximum and minimum; ****p<0.0001, Student's *t*-test; *n* = 54). C) Immunostained cells showing the cellular distribution of α_5_-integrin (red), α_v_β_3_-integrin (green) and vinculin (blue). Blue and yellow insets are 4× enlargements of corresponding boxes.

To confirm the microtubule-dependent changes in AC composition observed by MS, ACs were isolated from vehicle- and nocodazole-treated cells, and a subset of proteins identified from MS analysis assessed by Western blotting (Fig. 8a). Consistent with MS results, α_v_-integrin, tensin-1 and filamin A showed a substantial increase in abundance upon nocodazole treatment; the proteins ELKS (ERC1) and PDLIM5 showed a marginal increase. In addition, quantification of the area of the cell containing α_5_-integrin-, α_v_β_3_-integrin- and vinculin-containing ACs using immunostaining showed that there was no significant change to α_5_-integrin area upon nocodazole-treatment, whereas the α_v_β_3_-integrin and vinculin area increased approximately 5.5-fold and 2.0-fold respectively upon nocodazole-treatment (Fig. 8b).

Taken together, these results are in agreement with previous studies that showed the assembly and growth of focal adhesions upon the loss of microtubules [20], [24], [25]. In addition, the data extend these studies by characterising the changes to ACs in a global, unbiased manner and suggesting a modulation of AC components that is independent of integrin receptor.

### Changes to the maturation state of ACs upon microtubule disruption

In vehicle-treated cells, α_5_-integrin, α_v_β_3_-integrin and vinculin were found at the cell periphery in large focal adhesions, but α_5_-integrin was also found in centrally located streak-like structures that resembled fibrillar adhesions and other punctate AC structures. In nocodazole-treated cells, there was an increase in α_v_β_3_-integrin staining at the cell periphery in large focal adhesions, and α_5_-integrin and vinculin were also found at centrally located focal adhesion-like structures (Fig. 8c). These results suggest that microtubule disruption causes a switch in the maturation state of ACs towards focal adhesions, such that α_5_-integrin is redistributed to focal adhesions and is accompanied by an increase in the abundance of AC components.

**Figure 9:**
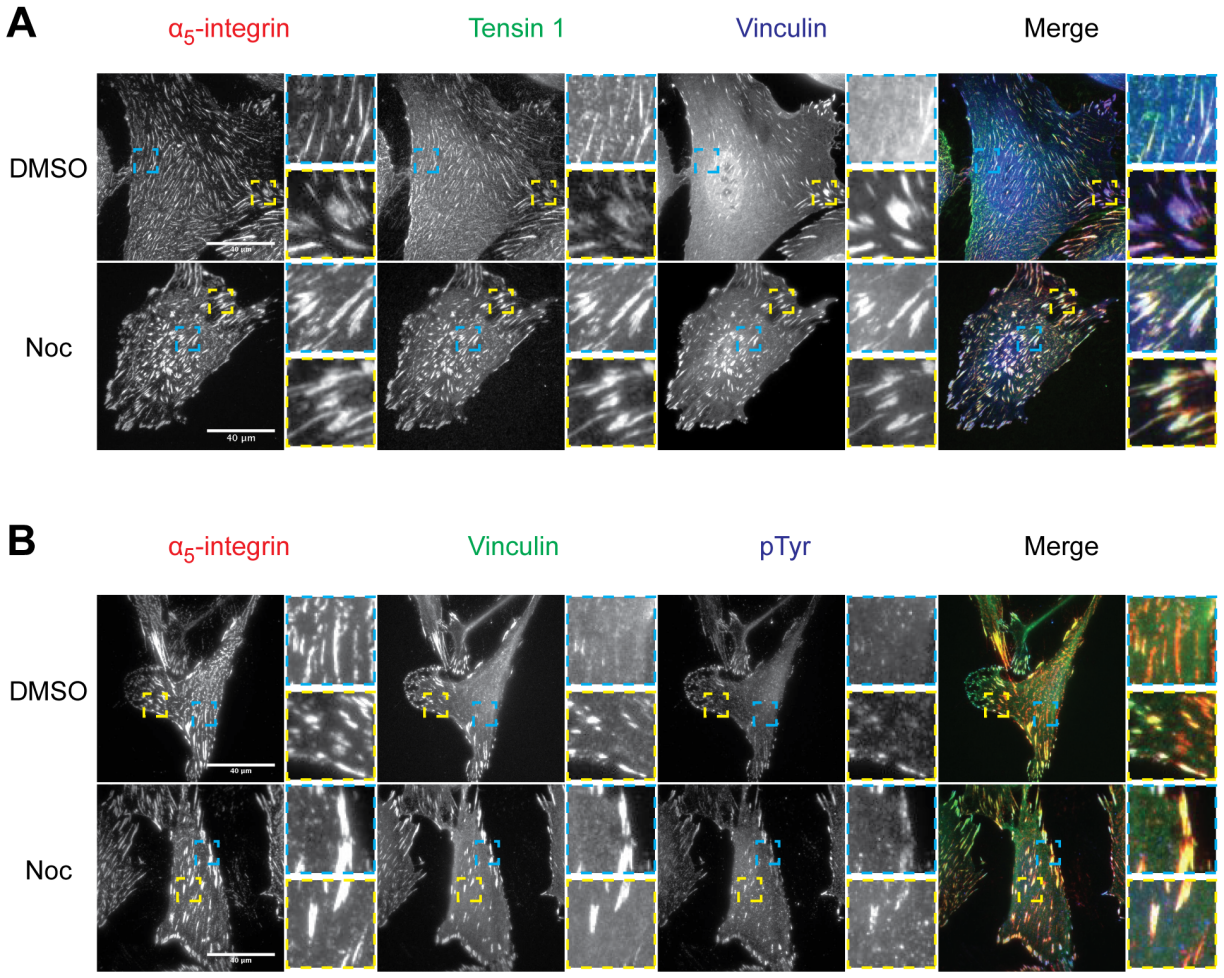
Changes to the maturation state of ACs upon microtubule disruption. Serum-starved HFFs were treated with DMSO and nocodazole (Noc) for 4 hours, fixed and immunostained for A) α_5_-integrin (red), tensin-1 (green) and vinculin (blue) and B) α_5_-integrin (red), vinculin (green) and pTyr (blue). Blue and yellow insets are 4× enlargements of corresponding boxes.

**Figure 10:**
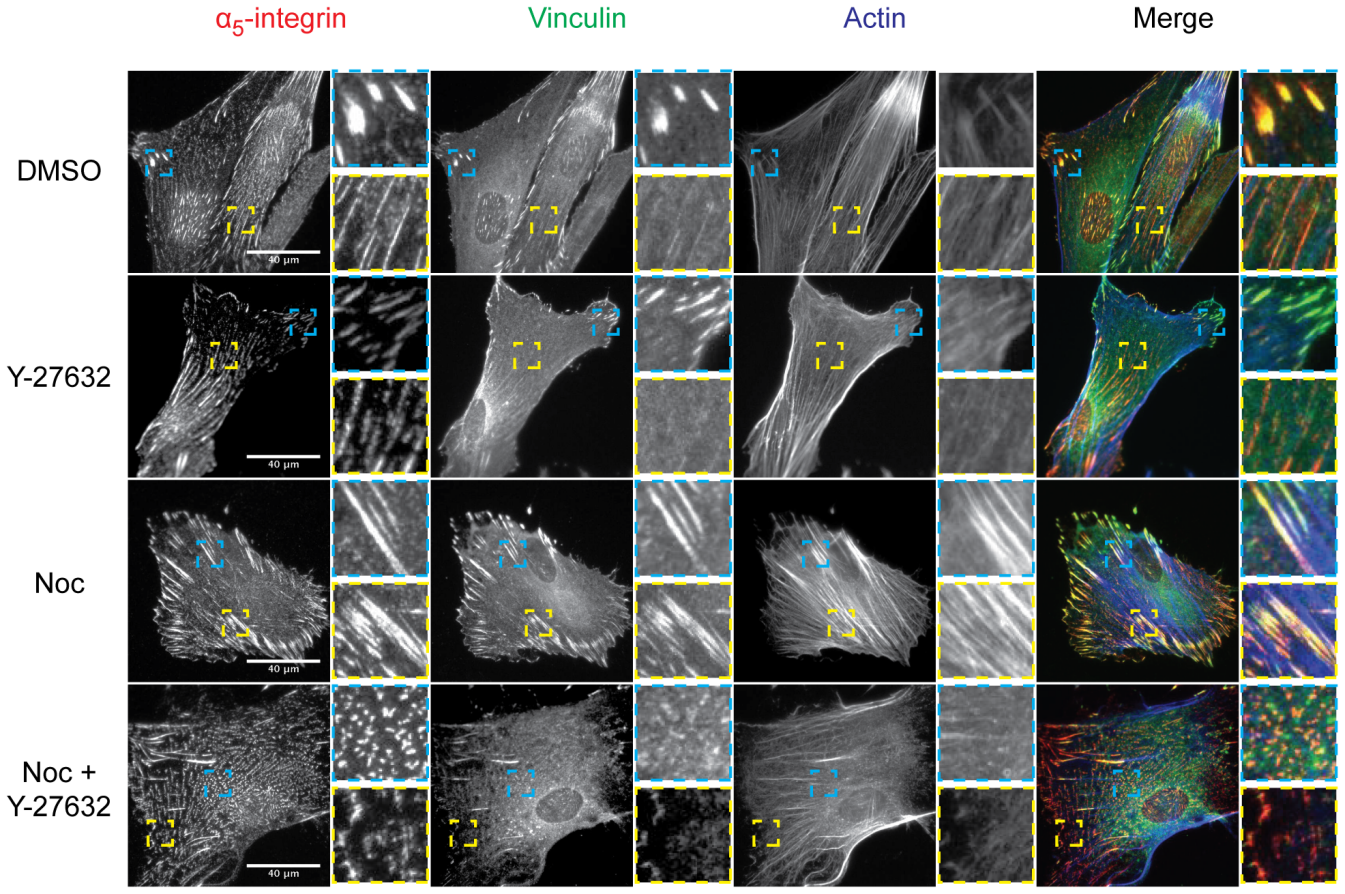
Force-dependence of the microtubule-induced AC modulation. Serum-starved HFFs were treated with DMSO, Y-27632, nocodazole (Noc) or a combination of Noc and Y-27632, fixed and immunostained for α_5_-integrin (red), vinculin (green) and actin (blue). Blue and yellow insets are 4× enlargements of corresponding boxes.

Fibrillar adhesions are involved in FN fibrillogenesis [42], [43], and are typically characterised by high-levels of α_5_β_1_-integrin and tensin, low-levels of paxillin, vinculin and pTyr [19], [21], and lack attachment to actin stress fibres [19], [44]. To determine if the adhesion structures in Fig. 8 were fibrillar adhesions and to investigate their modulation upon the loss of microtubules, vehicle- and nocodazole-treated cells were immunostained for tensin-1, pTyr (Fig. 9) and actin stress fibres (Fig. 10). In vehicle-treated cells, tensin-1 co-localised with α_5_-integrin throughout the cell, including the centrally located streak-like α_5_-integrin structures that displayed low levels of vinculin (Fig. 9a), whereas pTyr and thick actin stress fibres co-localised with peripheral α_5_-integrin ACs that had high levels of vinculin (Figs. 9b and 10). Therefore, these analyses indicate that the observed centrally located streak-like α_5_-integrin structures were fibrillar adhesions.

In nocodazole-treated cells, α_5_-integrin, tensin-1 and vinculin co-localised throughout the cell, at peripherally and centrally located focal adhesions (Fig. 9a). In addition, pTyr and thick actin stress fibres co-localised with peripheral and central α_5_-integrin structures that had high levels of vinculin (Figs. 9b and 10). Therefore, these results demonstrate that the loss of microtubules results in an increase in focal adhesions, particularly at the central regions of the cell, and a decrease in fibrillar adhesions and other AC structures. Taken together, these results further suggest that microtubule disruption causes an alteration in the maturation state of ACs to favour the formation of focal adhesions from other α_5_-integrin AC structures.

### Determining the force-dependence of the microtubule-induced AC modulation

Loss of cellular microtubules is accompanied by increased actomyosin contractility due to the release and activation of microtubule-localised GEF-H1, and the subsequent stimulation of RhoA [22], [23]. Therefore, to determine if the redistribution of α_5_-integrin and the alteration of the maturation state of ACs was due to microtubule-dependent changes in cellular force, Y-27632, a Rho-associated protein kinase (ROCK) inhibitor was used (Fig. 10). Cells treated with Y-27632 displayed sparse actin stress fibres, small peripheral vinculin-containing focal complexes, and centrally located α_5_-integrin fibrillar adhesions. In contrast, cells treated with nocodazole displayed thick actin stress fibres, large central and peripheral vinculin-containing focal adhesions. Co-treatment of cells with Y-27632 and nocodazole reversed the effects of nocodazole as cells displayed sparse actin stress fibres, small peripheral vinculin-containing focal complexes, and centrally located α_5_-integrin fibrillar adhesions. These data indicate that the redistribution of α_5_-integrin and the alteration of the maturation state of ACs to focal adhesions upon microtubule disruption is force-dependent and mediated through the RhoA-ROCK mediated pathway. In summary, these results demonstrate a complex interaction between microtubule dynamics, force generation and AC maturation state.

## Discussion

The microtubule network plays an important role in regulating ACs and previous studies have showed that microtubule disruption results in focal adhesion and actin stress fibre formation [20], [24], [25], [45]. To characterise the interplay between microtubules and ACs further, we employed a ventral plasma membrane isolation strategy and a quantitative MS-based proteomics approach to define the compositional changes in ACs upon microtubule disruption in a global, unbiased manner. We present a novel AC component MS dataset that describes the changes to ACs upon microtubule disruption. Our major finding is that, upon the loss of microtubules, the majority of AC components were increased in abundance at sites of focal adhesion formation, but the abundance of α_5_- and β_1_-integrins did not change. Further investigations revealed that this was due to an intracellular force-dependent switch in the maturation state of ACs towards focal adhesions, such that α_5_-integrin is redistributed to focal adhesions and is accompanied by an increase in the abundance of AC components. Therefore, this MS dataset catalogues the modulation of AC composition upon increased cellular force following microtubule disruption, and is complementary to previous studies that determined the force-dependent changes to AC composition upon myosin II inhibition with blebbistatin [33], [34], [46]. These data underscore the importance of microtubules in regulating intracellular forces to determine the different states of ACs and provide a resource for future studies of adhesion signalling.

In the basal resting state of a cell, a continuum of AC states exists that include nascent adhesions, focal complexes, focal adhesions and fibrillar adhesions [47], [48]. These different AC states not only differ in their size and location, they also differ in their composition and function [21], [42], [47], [48]. This study provides several lines of evidence to support a microtubule disruption-induced switch in AC states to favour large focal adhesions. Specifically, 1) the abundance of α_5_- and β_1_-integrins did not change; 2) the majority of AC components, particularly structural proteins, were increased; 3) the formation of numerous large focal adhesions at the periphery and even at central regions of cells; and 4) the reduction in other AC structures such as fibrillar adhesions.

The switch in AC state to favour focal adhesions can be attributed to the release and activation of microtubule-bound GEF-H1 and subsequent RhoA activation [22], [23]. RhoA acts on two distinct but concomitant pathways that are involved in focal adhesion maturation: mDia and ROCK [49], [50]. The activation of the RhoA-mDia pathway results in stress fibre formation [51], [52], whereas the RhoA-ROCK pathway promotes myosin II-mediated cell contractility [53]. Previous studies have implicated cell contractility in the formation of focal adhesions upon microtubule disruption [20]. To advance these studies, we have shown that increased cell contractility upon microtubule disruption occurs predominantly through the RhoA-ROCK-myosin II pathway, as specific inhibition of ROCK prevented focal adhesion formation upon microtubule disruption. Therefore, ACs respond to the increase in intracellular tension by reinforcing ACs with tension-sensitive proteins such as vinculin and talin. Indeed, it has been shown that upon nocodazole treatment, ACs are elongated centripetally, with vinculin being enriched at the distal ends of ACs nearest to actin stress fibres [25]. In agreement with these findings, we found an overall increase in abundance in structural AC proteins that link integrins to the actin cytoskeleton.

In our PPI network, we found many structural AC proteins that were highly connected and close in interaction network distance to the plasma membrane integrins. It has been suggested that the importance of a protein node to the maintenance of a protein interaction network is related to the density of its interactions [3]. PPI analysis revealed that proteins with the most interactions in the cytoplasm were reported to interact more directly with plasma membrane integrins (1-hop and 2-hops from integrins). Notably, the majority of highly interconnected (≥3 interactions) cytoplasmic proteins showed an increase in abundance upon the loss of microtubules. Taken together, these results suggest that the mechanosensing module involved in determining the nature of AC states upon microtubule disruption is likely to consist of structural proteins directly or closely bound to the integrins.

The loss of fibrillar adhesions coincided with the formation of large focal adhesions at the central regions of the cell upon microtubule disruption, thereby suggesting a switch from fibrillar adhesions to focal adhesions. This is surprising because it is generally thought that the maturation of ACs occurs in a unidirectional manner from nascent adhesions to focal complexes to focal adhesions and then to late-stage fibrillar adhesions. During the formation of fibrillar adhesions, α_5_β_1_-integrin bound to a deformable FN matrix is translocated centripetally [44]; therefore, the transmission of actomyosin contractility to the FN matrix via α_5_β_1_-integrin allows early events of FN fibrillogenesis to occur [42]. Fibrillar adhesions are structures with low tension [19], and inhibiting actomyosin contractility does not affect the presence of fibrillar adhesions, but prevents the formation of FN fibrils [19], [42]. Interestingly, inhibiting the pliability of the FN matrix by cross-linking inhibits the formation of fibrillar adhesions and FN fibrils, and promotes the formation of focal adhesion-like complexes containing α_5_β_1_-integrin, vinculin and paxillin [44]. This finding implies that when actomyosin forces are localised at ACs, tension-sensitive modules are recruited. Therefore, the conversion of fibrillar adhesions to focal adhesions upon microtubule disruption could be due to increased localised actomyosin forces experienced by α_5_β_1_-integrin complexes and the subsequent recruitment of tension-sensitive modules. Taken together, these findings suggests that the maturation state of ACs can occur bi-directionally and may allow the cell to regulate a response to intracellular or extracellular forces dynamically.

Previous studies using MS-based proteomics to define compositional changes in ACs have focused on the global changes to the adhesome upon myosin II inhibition using blebbistatin [33], [34]. Our findings are complementary to these studies because the loss of microtubules is accompanied with an increase in myosin II-stimulated actin contractility. It has been reported that a reduction in LIM domain-containing proteins is a major consequence of blebbistatin treatment [33]. Likewise, we show that LIM domain-containing proteins are increased upon nocodazole treatment. In addition, a recent study by Schiller *et al.* [46] delineated the different roles of β_1_- and α_v_-class integrins in rigidity sensing, and showed that β_3_-integrin focal adhesions are tension-dependent structures that recruit GEF-H1 and stimulate the formation of thick actin stress fibres, possibly through the RhoA-mDia pathway. Indeed, we found that microtubule disruption caused an increase in α_v_β_3_-integrin focal adhesions attached to thick actin stress fibres, which could be due to a combination of increased cell contractility, and increased availability of GEF-H1 to bind to β_3_-integrin tails. In addition, analysis of the MS dataset identified a cluster of proteins, consisting of ELKS, liprin α1 and liprin β1, that was enriched to ACs upon microtubule disruption. Recently, it has been shown that ELKS and liprin, along with LL5β, KANK1 and KIF21A, form cortical microtubule attachment complexes that tether microtubules to the plasma membrane via CLASPs [54], [55], and direct microtubules to sites around ACs to facilitate their disassembly, along with ECM degradation [30]. In agreement with these studies, we show that ELKS, liprin α1 and liprin β1 are enriched to isolated ACs upon nocodazole treatment. Interestingly, both LL5β and ELKS did not change significantly upon blebbistatin treatment [33], suggesting a microtubule-dependent, but not force-dependent, regulation of the localisation of this cluster of proteins. Taken together, these findings indicate that this MS dataset is a useful resource that describes the tension-sensitive and microtubule-dependent changes to ACs upon microtubule disruption.

In summary, we have used MS-based proteomics to define the compositional changes to ACs upon the loss of microtubules in an unbiased, global manner. This dataset is important as an exploration of the intricate relationship between microtubules, ACs, intracellular contractile force and the ECM. In addition, the dataset is complementary to previously published datasets [33], [34], [46], and provides a platform for hypothesis generation and future work exploring the interaction between microtubules and ACs.

## Supporting Information

S1 Fig **Western blots from** **Fig. 3** **.**
(TIF)

S2 Fig **Western blots from** **Fig. 8** **.**
(TIF)

S1 Table **MS data of FN DMSO, FN Noc, PDL DMSO, and PDL Noc.**
(XLSX)

S2 Table **Identified proteins and functional enrichment analyses.**
(XLSX)
