# Supplementary figures and images for: Microtubule-Dependent Modulation of Adhesion Complex Composition
Source: PLoS One. 2014 Dec 19;9(12):e115213. doi: 10.1371/journal.pone.0115213 (PMC4272306; doi:10.1371/journal.pone.0115213)

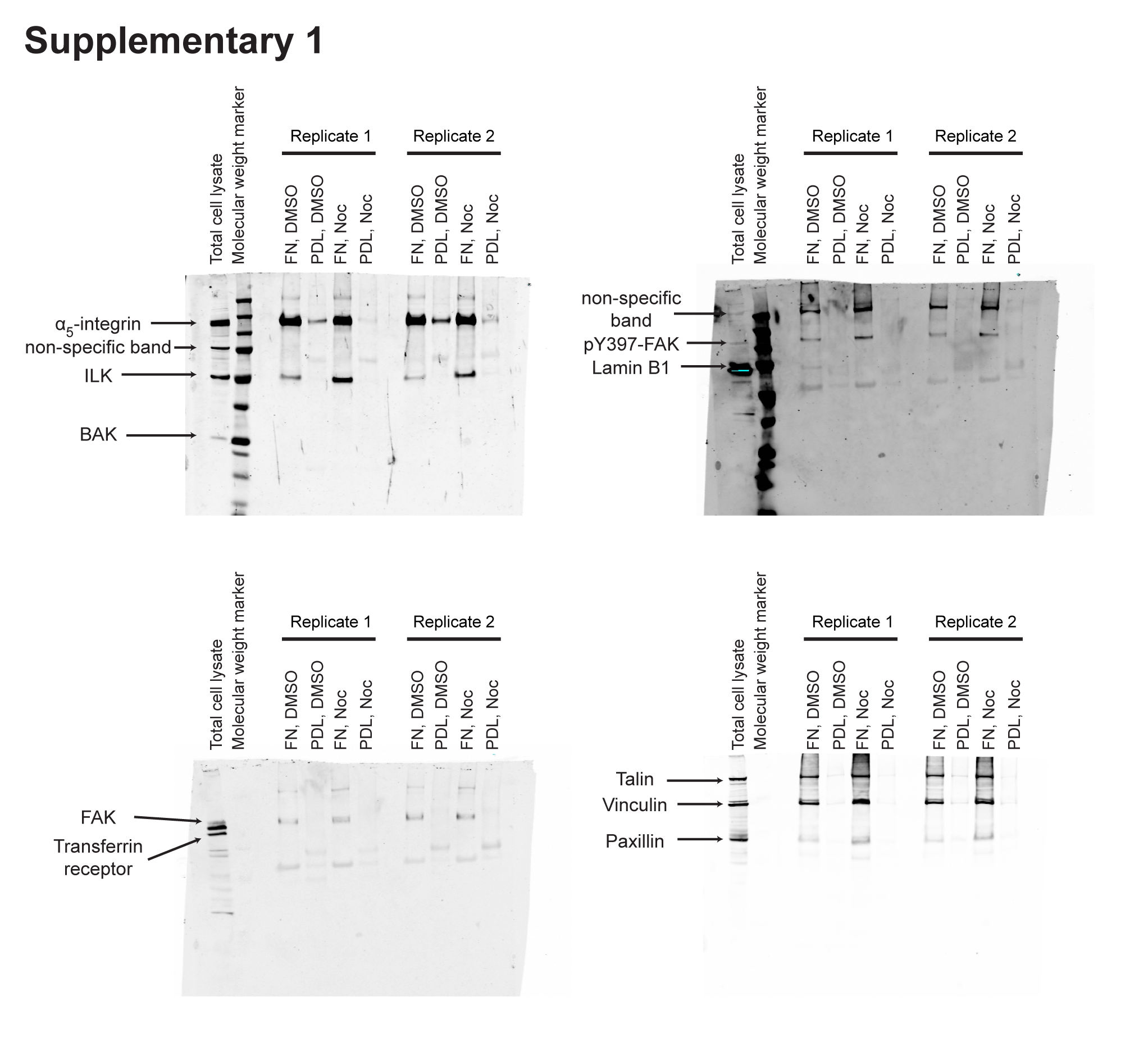

Supplement: S1 Fig — Western blots from Fig. 3 . (TIF) [file pone.0115213.s001.tif]

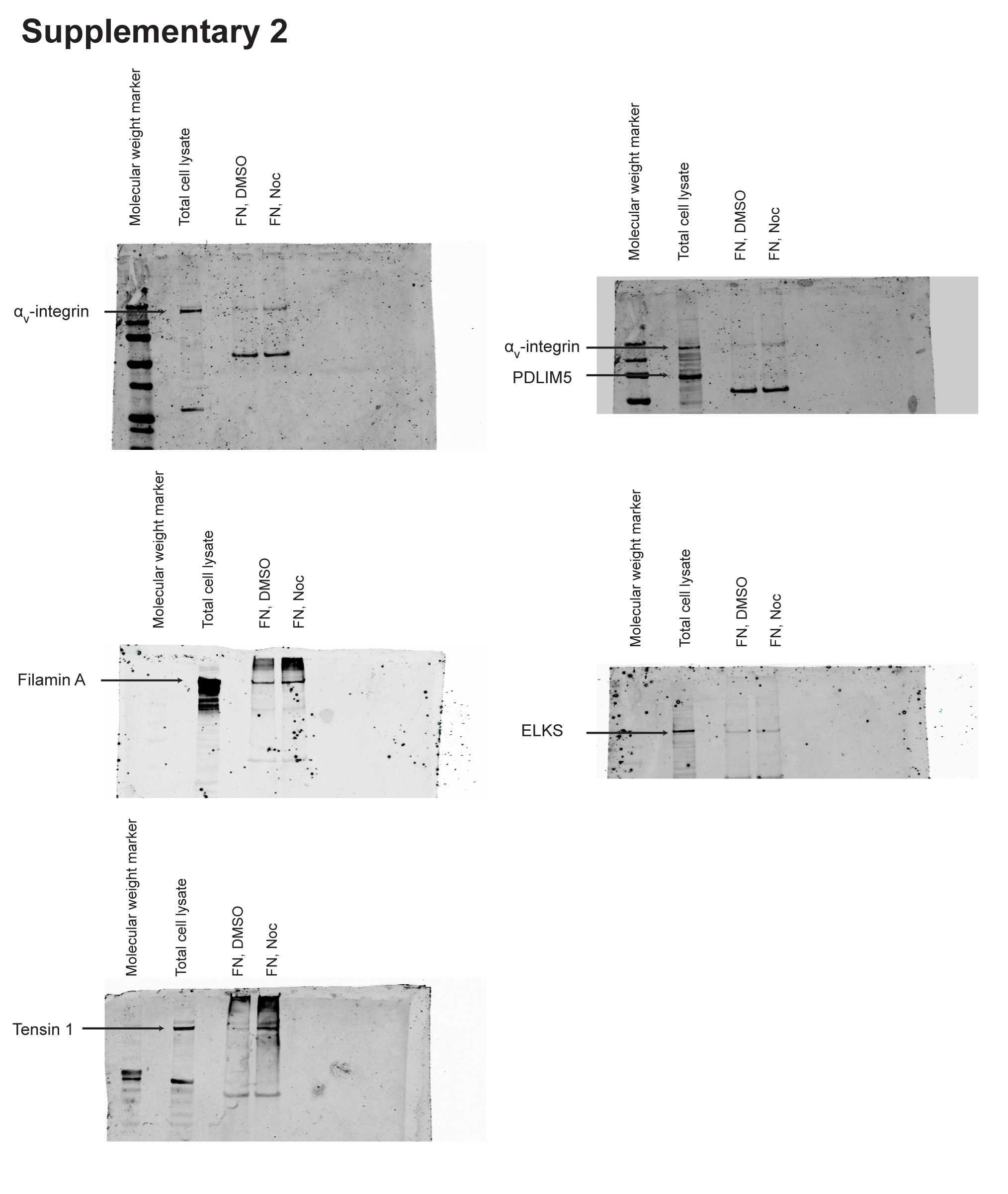

Supplement: S2 Fig — Western blots from Fig. 8 . (TIF) [file pone.0115213.s002.tif]
